# Point‐of‐care fluorescence imaging to optimise wound bed preparation prior to cellular and/or tissue‐based product (CTP) application

**DOI:** 10.1111/iwj.14446

**Published:** 2023-10-17

**Authors:** Thomas E. Serena, Keith Harding, Douglas Queen

**Affiliations:** ^1^ SerenaGroup® Research Foundation Cambridge Massachusetts USA; ^2^ School of Medicine Cardiff University Cardiff UK; ^3^ Medicalhelplines.com Inc Toronto Ontario Canada

My son's interpretation of ‘MAC Attack’ differs drastically from mine. His comes from a ravenous urge for two all‐beef patties on a sesame seed bun, whereas I am referring to one of the endless limitations placed on my practice by Medicare administrative contractors (MACs). The most recent ‘MAC Attack’ by Novitas, First Coast Service Options and CGS, representing 14 US states, restricted the use of Cellular and/or Tissue‐Based Products (CTPs), also known as ‘skin substitutes’, to four applications per diabetic foot or venous leg ulcer.^1^ These new local coverage determinations (LCDs) are effective as of 17th September 2023. Failure to achieve success after four CTP applications will result in the loss of patient access to this therapy—both ‘MAC Attacks’ have potential adverse health effects. But before villainizing the MAC medical directors for their decision, a review of the literature is warranted. In truth, CTPs used without proper wound bed preparation often fail. In fact, the results are worse than if a wound was never treated with a CTP.^2^

The key to success in healing wounds with CTPs is wound bed preparation: debridement, control of bacterial burden, maintaining an appropriate moisture balance, off‐loading for diabetic foot ulcers and compression for venous leg ulcers. Reduction of bacterial load is crucial prior to the application of CTPs; however, wound care specialists often rely solely on clinical signs and symptoms to detect bacterial load. The ability to detect clinically significant levels of bacteria using examination is poor (sensitivity less than 15%).^3^ Wound cultures are equally inaccurate,^4^ and it takes days for the results to return. The national and local coverage determinations for CTP use have required control of bacterial burden as a condition for reimbursement for years; however, clinicians have used and continue to use inaccurate and unreliable methods for determining bacterial load. The decision on when to apply a CTP is at best haphazard. The fault of this ‘MAC Attack’ lies with the wound care community and the slow adoption of diagnostics.

The most studied diagnostic in the detection of bacteria in acute and chronic wounds is fluorescence imaging—a point‐of‐care, non‐invasive, modality that safe uses violet light to detect bacterial loads greater than 10^4^ CFU/g.^3^ At this level, bacteria elicit changes at the cellular level that result in tissue damage and healing arrest, often without exhibiting signs of overt infection. This number of bacteria was recently termed chronic inhibitory bacterial load (CIBL).^5^ Clinical signs and symptoms of bacterial load are inaccurate in non‐healing wounds and are often completely absent in immunocompromised patients.^3^ The ability to detect a clinically significant level of bacteria, CIBL, prior to the application of a CTP using only signs and symptoms of infection is poor; however, fluorescence imaging can improve the detection of bacteria by fourfold to sevenfold^3^ irrespective of clinical expression and throughout all skin tones.

Despite being an accurate, bedside method of bacterial detection, the adoption of fluorescence imaging has been slow. Real‐time fluorescence imaging has been reported to improve both CTP and autologous skin grafting outcomes based on the presence or absence of bacterial fluorescence prior to application.^6^, ^7^ Further, fluorescence imaging improves reduction of bacterial burden, as demonstrated in numerous publications, by accurately identifying and localising high bacterial loads and guiding clinicians in the process of removing bacteria in real time.^8^, ^9^ A randomised control trial found that the most common intervention prompted by fluorescence imaging was debridement, and patients who received the imaging intervention showed improved healing (twice as many DFUs healed at 12 weeks than the standard of care).^10^

Wound specialists must expand their toolkit to optimise wound bed preparation prior to the application of CTPs or face stricter and broader restrictions. Fluorescence imaging in combination with physical examination enhances the identification of bacteria, which in turn improves wound healing outcomes with CTPs. Finally, as the wound care community builds a robust body of evidence on the optimisation of CTPs, the evidence garnered can be used to assuage the next ‘MAC attack.’
