# Corrigendum to point‐of‐care fluorescence imaging to optimize wound bed preparation prior to cellular and/or tissue‐based product (CTP) application

**DOI:** 10.1111/iwj.14497

**Published:** 2023-11-14

Serena TE, Harding K, Queen D. Point‐of‐care fluorescence imaging to optimise wound bed preparation prior to cellular and/or tissue‐based product (CTP) application. *Int Wound J*. 2023;20:3441–3442. https://doi.org/10.1111/iwj.14446

The sentence 4 and 5 “These new local coverage determinations (LCDs) are effective as of 17th September 2023. Failure to achieve success after four CTP applications will result in the loss of patient access to this therapy—both ‘MAC Attacks’ have potential adverse health effects.” was incorrect.

This should have read: “Proposals to restrict the number of Skin Subs/CTP applications have ignited fervent debates within Medicare over the past few years, leaving policymakers unsure about how best to address this issue. These…. MAC attacks.”

The below paragraphs should have been added at the end of the article.

IWJ Editorial Comment.

We are publishing the editorial by Dr Serena in this edition of the IWJ in an attempt for us to address important developments and controversies in the field. For many of us, we do not have the benefit of access to the cell‐based therapies that are used in the United State of America so the editorial has little impact on our practice. We publish the article to show how decisions made by outside bodies and reimbursement authorities can have a huge impact on clinical practice.

We feel it is important for readers of the IWJ to hear about such matters to become aware of consider what they might see as a way of addressing challenging issues that may impact their practice. We do hope this editorial will spark further letters to the editor on controversial topics. We want to hear your thoughts and ideas to ensure the journal is allowing such matters to be debated and shared with our readers. Please engage with us.

Professor Keith Harding, Editor‐in‐Chief, IWJ.

We apologize for this error.

